# Supplementary material for: Treatment with oxfendazole increased levels of cardiac troponin I in pigs naturally infected with Taenia solium cysticercosis
Source: PLoS One. 2025 May 8;20(5):e0321735. doi: 10.1371/journal.pone.0321735 (PMC12061117; doi:10.1371/journal.pone.0321735)
Supplement: S1 Table — (PDF) [file pone.0321735.s001.pdf]

S2 File. Treatment with Oxfendazole increased levels of cardiac troponin I in pigs naturally infected with *Taenia solium* cysticercosis.

Table 2. Summary of descriptive statistics of serum cardiac troponin I (cTnI). These were samples collected from adult pigs that were both positive [IT= infected and treated (n=9), INT= infected and not treated (n=8)] and negative [NINT= not infected and not treated (n=9) and NIT= not infected and treated (n=9)] for *T. solium* cysticercosis following treatment with oxfendazole at 30mg/kg effective dose. The concentration of cTnI was measured from day 0 (before treatment) to 11 weeks post treatment.

TREATMENT GROUP	STATISTIC								
	**Min**	**25%-percentile**	**Median**	**75%-percentile**	**Max**	**Mean**	**Standard deviation**	**Lower 95% CI**	**Upper 95% CI**
**NIT *(n= 9)***	0.004	0.010	0.015	0.020	0.040	0.016	0.007	0.014	0.017
**NINT *(-ve control) (n= 9)***	0.003	0.010	0.013	0.018	0.033	0.014	0.006	0.012	0.015
**INT *(+ve control) (n= 8)***	0.002	0.014	0.021	0.028	0.070	0.024	0.015	0.0.021	0.027
**IT *(n= 9)***	0.003	0.013	0.021	0.046	0.367	0.041	0.051	0.031	0.051
