# Supplementary material for: Treatment with oxfendazole increased levels of cardiac troponin I in pigs naturally infected with Taenia solium cysticercosis
Source: PLoS One. 2025 May 8;20(5):e0321735. doi: 10.1371/journal.pone.0321735 (PMC12061117; doi:10.1371/journal.pone.0321735)
Supplement: S3 File — (PDF) [file pone.0321735.s004.pdf]

cystinet africa
Cysticercosis and Taeniosis Network of Sub-Saharan Africa

STANDARD OPERATING PROCEDURE

PIG DISSECTIONS

- All cysticercosis infected pigs (Treated and Not treated i.e. IT and INT) will be selected for dissection. All selected pigs will be entered into an excel sheet.
- Fill in dissection form “pig dissection sheet’, one form for each pig

PIG DISSECTION SHEET

DATE	NAME VILLAGE	NAME HH HEAD	HH CODE	pig age	M / F	code serum	start time:
__/__/___							end time:

TP pos □ neg □	LOCALISATION & NUMBER:in categories: <10; 10-50; 50-100; >100										
T. solium STAGE CYST	masseter	heart	tongue	psoas	diafragm	oesophagus	front legs	hind legs	liver	other	TOTAL
viable											
degenerated											
calcified											
TOTAL											
T. hydatigena											
Other											

Cysts collected for PCR?	Y / N	if yes, code: ________	n° of vials: _____
Nr of blood tubes collected:	____ of __ml		

Comments:

Name & signature

PIG DISSECTIONS

- Wear gloves and protective clothing, including facial mask

- When euthanizing: collect blood,

✓ Collect serum samples: collect blood in falcon tubes (?): but different centrifuge, complicated in the field? We don’t want to lose these documented sera? …work with 50 ml and no spinning? Or 15ml if fit in normal centrifuge?

- Check tongue: visual & palpate for cysts:

- Cut all meat and organs in 5mm slices, start with predilection sites:

1. heart

2. masseters
3. tongue
4. Brain

- Always also slice the liver

- Write down: tick number of cysts detected, the aim is to have categories:<10 cysts; 10-50 cysts; 50-100 cysts; >100 cysts
- ✓ Indicate viable-degenerated – calcified

- All sliced (infected?) meat/organs should go in bin bags and should be burned (?)
- Disinfect the working space with water and detergent

PIG DISSECTIONS
