# Supplementary material for: Treatment with oxfendazole increased levels of cardiac troponin I in pigs naturally infected with Taenia solium cysticercosis
Source: PLoS One. 2025 May 8;20(5):e0321735. doi: 10.1371/journal.pone.0321735 (PMC12061117; doi:10.1371/journal.pone.0321735)
Supplement: S4 File — (PDF) [file pone.0321735.s005.pdf]

ULTRA-SENSITIVE PIG CARDIAC TROPONIN-I ELISA
Life Diagnostics, Inc., Cat. No. CTNI-9-US

INTRODUCTION

Cardiac Troponin-I (CTNI) is a component of the troponin complex that regulates muscle contraction. After cardiac injury, CTNI is released into the blood. Because it is expressed specifically in the heart it is an excellent biomarker of cardiac injury. In humans, CTNI levels peak 12-24 hours after injury, returning to baseline within 2-6 days. In mice, levels peak as early as 1 hour and return to normal within 1-3 days.[1] Our ELISA kits have been used extensively since 2003 for measurement of CTNI in all species used in preclinical research. A representative list of publications citing their use can be found on our cardiac biomarker ELISA kits webpage https://lifediagnostics.com/cardiac-biomarker-elisa-kits/

PRINCIPLE OF THE ASSAY

The ELISA uses two different antibodies that recognize a relatively protease-resistant epitope on CTNI. One is used for solid phase immobilization (microtiter wells). The second is conjugated to horse radish peroxidase (HRP) and used for detection. Samples (serum or plasma) and standards (200 µl) are pipetted into the microtiter wells and incubated for 2 hours on a plate shaker. After washing the wells, 100 µl of diluent and 100 µl of HRP-conjugate is pipetted into the wells. The plate is incubated for one hour. During this step, if CTNI is present, it is sandwiched between the immobilization and HRP-conjugated antibodies. The wells are then washed to remove unbound HRP-conjugate. TMB is added and incubated for 20 minutes. If CTNI is present a blue color develops. Color development is stopped by addition of Stop solution, changing the color to yellow. Absorbance is measured at 450 nm. The concentration of CTNI is proportional to absorbance and is derived from a standard curve.

MATERIALS AND COMPONENTS

Materials provided with the kit:

- Anti-CTNI coated plate (12 x 8-well strips)
- CTNI Stock
- Diluent: YD25-1, 25 ml
- HRP Conjugate, 11 ml
- 20x Wash solution: TBS50-20, 50 ml
- TMB: TMB11-1, 11 ml
- Stop solution: SS11-1, 11 ml

Materials required but not provided:

- Pipettors and tips
- Distilled or deionized water
- Polypropylene or glass tubes
- Vortex mixer
- Absorbent paper or paper towels
- Plate incubator/shaker
- Plate washer
- Plate reader capable of measuring absorbance at 450 nm.
- Curve fitting software

STORAGE CONDITIONS

Store the lyophilized stock at or below -20°C. The remainder of the kit should be stored at 4°C and the microtiter plate should be kept in a sealed bag with desiccant. Kits will remain stable for six months from the date of purchase.

GENERAL INSTRUCTIONS

1. All reagents should be allowed to reach room temperature before use.
2. Reliable and reproducible results will be obtained when the assay is carried out with a complete understanding of the instructions and with adherence to good laboratory practice.
3. The wash procedure is critical. Insufficient washing will result in poor precision and falsely elevated absorbance readings.
4. Laboratory temperature will influence absorbance readings. Our ELISA kits are calibrated using shaking incubators set at 150 rpm and 25°C. Performance of the assay at lower temperatures will result in lower absorbance values.

WASH SOLUTION PREPARATION

The wash solution is provided as a 20x stock. Prior to use, dilute the contents of the bottle (50 ml) with 950 ml of distilled or deionized water.

STANDARD PREPARATION

1. Reconstitute the lyophilized CTNI stock with de-ionized or distilled water as detailed on the vial label. Mix gently until dissolved.
2. Label 7 polypropylene tubes 1.0, 0.50, 0.25, 0.125, 0.0625, 0.0313 and 0.0156 ng/ml.
3. Into the tube labeled 1.0 ng/ml, pipette the volume of diluent detailed on the stock vial label. Then add the indicated volume of stock and mix gently. This provides the 1.0 ng/ml standard.
4. Pipette 250 µl of diluent into the tubes labeled 0.50, 0.25, 0.125, 0.0625, 0.0313 and 0.0156 ng/ml.
5. Prepare a 0.50 ng/ml standard by diluting and mixing 250 µl of the 1.0 ng/ml standard with 250 µl of diluent in the tube labeled 0.50 ng/ml. Similarly prepare the remaining standards by two-fold serial dilution.

The reconstituted CTNI stock should be frozen immediately after use. It remains stable when frozen or at least 1 month at -20°C and 6 months at -70°C. Discard the working standards after use.

SAMPLE COLLECTION AND PREPARATION

Serum or plasma should be prepared as quickly as possible after blood collection and stored at 4°C. All samples should be similarly processed (i.e., storage times and temperatures should be the same). If serum samples cannot be assayed immediately, they should be frozen at –70°C and thawed only once prior to use. Undiluted serum can be used with this kit. If dilution is necessary, use diluent YD25-1. Other diluents must not be used.

ASSAY PROCEDURE

1. Secure the desired number of 8-well strips in the holder. Unused strips should be stored in the re-sealed bag with desiccant at 4°C for future use.
2. Dispense 200 µl of standards and samples into the wells (we recommend that standards and samples be run in duplicate).[a]
3. Incubate on a plate shaker at 150 rpm and 25°C for 2 hours.

---

[a] 100 µl of sample may be used if volume is limiting. However, the volume of all other samples and standards used in the assay must also be 100 µl. Absorbance values will be slightly lower.

Life Diagnostics, Inc., PO Box 5205, West Chester, PA 19380
610-431-7707 – 610-431-7818 (Fax)
info@lifediagnostics.com – www.lifediagnostics.com

4. Empty and wash the microtiter wells 5x with 1x wash solution using a plate washer (400 µl/well).
5. Strike the wells sharply onto absorbent paper or paper towels to remove all residual droplets.
6. Add 100 µl of diluent to each well.
7. Add 100 µl of HRP-conjugate to each well.
8. Incubate on a plate shaker at 150 rpm and 25°C for one hour.
9. Empty and wash the microtiter wells 5x with 1x wash solution using a plate washer (400 µl/well).
10. Strike the wells sharply onto absorbent paper or paper towels to remove all residual droplets.
11. Dispense 100 µl of TMB into each well.
12. Incubate on a plate shaker at 150 rpm and 25°C for 20 minutes.
13. After 20-minutes, stop the reaction by adding 100 µl of Stop solution to each well.
14. Gently mix. It is important to make sure that all the blue color changes to yellow.
15. Read absorbance at 450 nm with a plate reader within 5 minutes.

CALCULATION OF RESULTS

1. Using curve fitting software, construct a standard curve by plotting absorbance values of the standards versus $\log_{10}$ of the concentration.
2. Fit the standard curve to a four-parameter logistic regression (4PL) equation (x axis = $\log_{10}$ concentration) and determine the concentration of the samples.
3. Multiply the derived concentration by the dilution factor (if applicable) to determine the actual concentration in the original sample.
4. If the A_{450} values fall outside the standard curve, samples should be diluted appropriately and re-tested.

TYPICAL STANDARD CURVE

A typical standard curve is shown below. This is for illustration only. A standard curve must be generated for each experiment.

CTNI (ng/ml)	A_{450}
1.000	2.405
0.500	1.265
0.250	0.730
0.125	0.452
0.0625	0.309
0.0313	0.264
0.0156	0.153

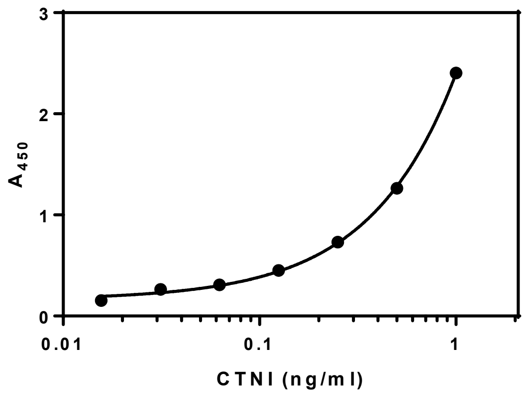

REFERENCES

1. Engle SK. et al. Qualification of cardiac troponin I concentration in mouse serum using isoproterenol and implementation in pharmacology studies to accelerate drug development. Toxicologic Pathology. 37:617-628 (2009)

Rev 10092020

For technical assistance please email us at
techsupport@lifediagnostics.com
