# Supplementary material for: Treatment with oxfendazole increased levels of cardiac troponin I in pigs naturally infected with Taenia solium cysticercosis
Source: PLoS One. 2025 May 8;20(5):e0321735. doi: 10.1371/journal.pone.0321735 (PMC12061117; doi:10.1371/journal.pone.0321735)
Supplement: Attachment — Submitted filename: Response to Reviewers.pdf [file pone.0321735.s007.pdf]

The University of Zambia
School of Veterinary Medicine
P.O. Box 32379
Lusaka, Zambia

20th September, 2024

The Editor in Chief,
PLOS One

Dear Sir/Madam,

REF: RESPONSE TO REVIEWER COMMENTS.

I am writing to respond to the reviewer's comments on our manuscript, **"Treatment with Oxfendazole increased levels of cardiac troponin I in pigs naturally infected with *Taenia solium* cysticercosis."** We appreciate the reviewer's thoughtful feedback, which has significantly improved the quality of our work. We have addressed each comment and observation noted by each review as outlined below:

REVIEWER #1 (PONE-D-24-05361)

Overall Comments:

"The study lacks novelty, since it is already expected that anthelmintic treatment with OFZ damages cysts located in cardiac muscle, and activates the host's immune system to produce a strong inflammatory response (several studies have demonstrated this using more precise markers such as histopathology or immunohistochemistry)."

"It is not known and not well detailed why studying cTn1 may help to a better understanding of the immunopathological processes that occur in CC and NC."

Response: The basis of this study was on the understanding that animals such as pigs, mice, and guinea pigs for example, have long been used to study various diseases before such studies are done in humans e.g., clinical trials, basic experiments. In this particular case, cardiac troponin I (cTnI) is a biomarker indicative of cardiac damage in humans in various diseases/conditions. Therefore, the study aimed at finding out if this cardiac troponin I (cTnI) can also be used to indicate the same in pigs with cysticercosis after treatment. This is on the basis that pigs positive on tongue examination are heavily infested with cysts in the heart (cardiac muscle), among other organs as shown by several studies. Furthermore, treatment with an anthelminthic such as OFZ results in an inflammatory response that causes damage to the surrounding tissue and muscles.

References:

1. Iburg TM, Karlsson M, Spång F, Sikasunge CS, Johansen MV. The effect of oxfendazole treatment on muscle pathology in pigs infected with Taenia solium cysticercosis. Vet Parasitol. 2012 Dec 21;190(3–4):442–6.

2. Sikasunge CS, Phiri IK, Willingham AL, Johansen M V. Dynamics and longevity of maternally-acquired antibodies to Taenia solium in piglets born to naturally infected sows. Veterinary Journal. 2010 Jun;184(3):318–21.

3. Sikasunge CS, Johansen M V., Willingham AL, Leifsson PS, Phiri IK. Taenia solium porcine cysticercosis: Viability of cysticerci and persistency of antibodies and cysticercal antigens after treatment with oxfendazole. Vet Parasitol. 2008 Nov 25;158(1–2):57–66.

"A positive Ag-ELISA result may give false-positives, especially in pigs with low cyst burden (pigs with only a single cyst on tongue exam. ¿Pigs where randomized according to Ag-ELISA levels?"

Response: When selecting pigs for inclusion into the study, it was based on tongue examination for both positive and negative pools of pigs and additionally Ag-ELISA for the negative pigs in order to determine if these pigs are really negative in as much as they were purchased from cysticercosis free farms with good health records. See lines 168-173 of the revised manuscript.

"The discussion section lacks a paragraph of limitations/strengths of the study."

Response: The study limitations and challenges have been added in the discussion section of the manuscript. See lines 436-460 of the revised manuscript.

Specific comments

Line 28: "On cardiac troponin 1 (cTn1) elevation in ……"

Response: inserted 'elevation' in the text. See line 29 of the revised manuscript.

Lines 39-42: add P values for significance as required

Response: The missing p-values and means have been inserted as per reviewers' observations. See line 39-46 of the revised manuscript.

Lines 42-43: How you can determine this?

Response: several studies have shown that treatment of pigs with cysticercosis is followed by an inflammatory response which is reflected in this case by the rise in cTnI levels in the infected and treated group (IT) as has been demonstrated in this particular study.

References:

1. Iburg TM, Karlsson M, Spång F, Sikasunge CS, Johansen MV. The effect of oxfendazole treatment on muscle pathology in pigs infected with Taenia solium cysticercosis. Veterinary parasitology. 2012 Dec 21;190(3-4):442-6.
2. Mkupasi EM, Ngowi HA, Sikasunge CS, Leifsson PS, Johansen MV. Efficacy of ivermectin and oxfendazole against Taenia solium cysticercosis and other parasitoses in naturally infected pigs. Acta tropica. 2013 Oct 1;128(1):48-53.

Line 44: "This study shows that…."

Response: The statement 'This study shows that' has been inserted in the text. See line 47 of the revised manuscript.

Lines 62-64: Humans are not more propense to develop NCC than pigs. This occurs because cysts in the CNS survive for a longer time. Please, modify.

Response: The statement has been modified as per reviewer's suggestion to read as, "Cysticerci can also localize in the central nervous system where they survive for a longer-period of time, giving rise to neurocysticercosis (NCC) and inducing neurological signs and symptoms." See lines 67-69 of the revised manuscript.

Lines 92-94: Is not clear the link for studying cTn1 for cardiac damage in the context of porcine cysticercosis. Especially, not all pigs with cysticercosis have cysts in their heart. Justify.

Response: When selecting pigs for inclusion into the study, it was based on tongue examination for both positive and negative pools of pigs and additionally Ag-ELISA for the negative pigs in order to determine if these pigs are really negative in as much as they were purchased from cysticercosis free farms with good health records. Carcass dissections at the end of the study also confirmed presence of cysts in the heart. Furthermore, several studies have shown that pigs positive on tongue examination for cysticercosis are heavily infested with cysts in the heart (cardiac muscle), among other organs.

Line 134-135: "the role of cTnI on inflammation" or "cTnI as marker of heart damage/inflammation"??

Response: The sentence has been revised and inserted "cTnI as a marker of heart damage/inflammation." See lines 145-146 of the revised manuscript.

Lines 166-175: I consider this section as unnecessary (please, remove it).

Response: This manuscript was written according to the Arrive Guidelines 2.0 - Conducting Animal Research. These require information about housing and husbandry as well as animal care during the research. However, as per reviewers comments the "Washing and dipping" section has been deleted.

Lines 187-191: It is not described the dimensions of experimental corrals, how many animals per corral.

Response: The dimensions of the pig pens has been inserted within the text under biosecurity section as observed by the reviewers i.e., $20m^2$ (4m x 5m), with each pen housing 5 pigs. See lines 187-188 of the revised manuscript.

Lines 214-215: in fasting??

Response: Yes, this was done in fasted animals that had been fasted for 12 hours in order to allow the anthelminthic to have maximum effect. See line 214.

Lines 246-246: What happen if there were differences between duplicates (e.g., variation more than 50%)?

Response: In the case of large variations of more than 50%, the associated sample would have to be repeated or run again. In this particular study there were no large variations between duplicate samples of more than 50%. It's also worth noting that duplication of samples is done in order to reduce variation.

Lines 281-284: It would be useful to report how antigen levels were described during follow-up according experimental groups (mean ± SEM?).

Response: We appreciate the reviewer's comment. The antigen against *T. solium* were only used during recruitment of pigs into the study to confirm cysticercosis status besides tongue examination The focus was on the concentration of cTnI levels in cysticercosis pigs. However, cTnI levels in each treatment/control group have been described on an hourly and weekly basis during follow up.

Line 293: It would be interesting to describe cTnI levels in pigs among experimental groups at baseline (and baseline statistical comparisons).

Response: We appreciate the reviewer's suggestion and baseline concentrations and statistics have been included in the results section. See lines 306-308 of the revised manuscript.

Lines 293-298: You should emphasise the increased levels in cTn1 in the IT pigs versus the other groups.

Response: Inserted "versus other control and treatment groups," in the text as advised by the reviewer. See lines 308-309 of the revised manuscript.

Line 304: Figure 304: (n=9, n=9, n =…. looks very repetitive, please modify)

Response: The sample size has been modified and have only included the total number of pigs in the study i.e., n = 35. See line 322 of the revised manuscript.

Lines 313-316: It is not necessary to report the F test, and degrees of freedoms for ANOVA results, just P value.

Response: The F value and degrees of freedom have been deleted as advised. Only p-values have been reported. See line 331 of the revised manuscript.

Lines 314-315: It is not clear what the modified effect is. ¿Did you see the modification effect between experimental group in hourly and weekly observations? That should be the effect, since most of the difference between IT group versus and the other groups were observed until 72 hours after treatment, but not observed during weekly observations.

Response: This modification effect was seen in the hourly observations and not the weekly observations as observed by the reviewer. I have since inserted the following sentence in the text to read, "The effect of treatment was observed only in the IT group and this difference remained so until 72hrs post treatment and only to reach baseline levels at 1 week post treatment." See lines 331-333 of the revised manuscript.

Lines 317-326: Differences should emphasise that cTnI levels were higher for IT pigs (not just "different").

Response: As observed by the reviewer, the emphasis has been Inserted to read as "Furthermore, multiple comparisons between the treatment and control groups revealed that there was a higher cTnI concentration in the IT group in comparison to other treatment and control groups," in the text emphasizing the higher cTnI levels in the IT group. See lines 337-340 of the revised manuscript.

Lines 329-337: In the first paragraph of the discussion section, you describe again results using Mean ± SEM. It looks like results section again (in fact this should be in the results section). Re-paraphrase this section.

Response: This section has been revised and now reads, "The findings in this study provided valuable insights into the response of cardiac troponin I in both treated and untreated *T. solium* cysticercosis porcine model. This study revealed that treatment in pigs with cysticercosis had an effect on cTnI levels. Higher concentrations of cTnI were observed in the IT group in comparison to the other control and treatment groups. This observation underscores the significant impact of treatment on cTnI release in pigs with cysticercosis." See lines 370-375 of the revised manuscript.

Lines 338-342: This section of the discussion is not described or supported in the results section. The impact of antiparasitic treatment on acute inflammation (as soon as 48h after treatment onset) has been widely described in previous studies using the NCC pig model, cite them.

Response: This section has been reported in the results section as observed by the reviewer and now reads, "Hourly observations post-treatment revealed a significantly high concentration of cTnI in the IT group versus other control and treatment groups (Fig 4). This rise in concentration was noticed from 24hrs post treatment, with the maximum concentration of 0.115 ng/ml been attained 48hrs post treatment. The concentration of cTnI decreased rapidly to 0.062 ng/ml going into 72hrs and reaching baseline levels of 0.022 ng/ml 312 at one week post treatment. The NIT and NINT groups (negative pigs) did not show any significant

differences (p = 0.988), in terms of the cTnI concentrations, but were significantly lower than the infected groups." See lines 308-315. This section has since been revised in the discussion and the references added. See lines 376-385 of the revised manuscript.

Lines 358-361: the higher levels during weekly observation in the INT group compared to the IT group can also reflect differences in pre-existing cyst inflammation between groups using the natural pig model (a main drawback when using naturally infected pigs).

Response: The comment above from the reviewer has been added in the text i.e., "The higher levels during weekly observation in the INT group compared to the IT group can also reflect differences in pre-existing cyst inflammation between groups using the natural pig model, which is a main drawback when using naturally infected pigs. This is because these pigs pick up infection at different stages and its quite challenging to determine when they were infected." See lines 404-408 of the revised manuscript.

Lines 382-403: Conclusions are too long. You should focus on the main results and potential impact.

Response: The conclusion has been revised to only focus on main results and potential impact. See lines 464-471 of the revised manuscript.

REVIEWER #2 (PONE-D-24-05361)

Overall Comments:

"It is a very nice and well-prepared manuscript that reports the possible myocardial damage after treatment with oxfendazole in pigs naturally infected with Taenia solium. The paper is well organized and generally well written, though there are a few minor errors throughout the manuscript. The relevant literature is well-reviewed. Therefore, I suggest publishing this paper after revisions, primarily to address minor issues and correct a few stylistic errors."

"In the Materials and Methods section, it is crucial to specify whether the infected pigs were breed or not (I assume they weren't), and the same goes for the non-infected ones. The latter came from commercial farms; I assume they were not crossbred animals. Normally, pigs from technical farms display different physiological responses compared to crossbred animals (not breeds) and free-range animals, such as stress during weaning, increased nervousness, and different feeding methods. To avoid conjecture, you must provide specific details about pigs."

Response: As noticed by the reviewer, the naturally infected *T. solium* cysticercosis pigs (both treated and untreated) were not bred but purchased from the market upon positivity to *T. solium* cysticercosis on tongue examination, and these were free range pigs. On the other hand, the *T. solium* negative pigs were purchased from commercial farms and these were cross breeds. See lines 157-166 of the revised manuscript. However, as per reviewer's comments, pigs kept under free range and intensive systems display different physiological responses, which was one of

the limitations of this study and is now included within the discussion. See line 436-461 of the revised manuscript.

“Similarly, the use of naturally infected *T. solium*-infected pigs makes it crucial to determine the approximate age of the animals. This would indirectly help to determine the age of the cysts. Remember that older pigs are more likely to have degenerated cysts.”

Response: This is well noted. Age determination is a bit of a challenge especially in free range pigs and hence the reason for the inclusion criteria being size, height and weight of the pigs.

“This last observation prompts me to question why they didn't perform necropsies on the pigs to assess the status of the cysts in the untreated pigs. You would even have been able to evaluate myocardial damage through histopathology. You could potentially include this as one of the "limitations of the study" in the Discussion section.”

Response: Necropsies were conducted at the end of the study. And these have been added in the M&M and Results section. See line 227-234 and 349-367 of the revised manuscript. However, the only limitation was that the level of inflammation was not determined as this required slaughtering of pigs at each sampling point which would not have made any statistical sense in that there were only 9 pigs in each treatment/control group. However, study limitations have been included in the manuscript. See lines 453-457 of the revised manuscript.

Minor comments

Title: replace “Oxfendazole” with “oxfendazole”

Response: Replaced “Oxfendazole” with “oxfendazole” as per reviewer’s comments.

Line 44: replace “Taenia solium” with “T. solium”

Response: Replaced *“Taenia solium”* with *“T. solium”* as per reviewer’s comments. See line 47 of the revised manuscript.

Line 60: "metacestode larval stages" is redundant; it uses only "metacestode"

Response: Replaced "metacestode larval stages" with “metacestode” as per reviewer’s comments. See line 65 of the revised manuscript.

Line 61: humans are “accidental intermediate hosts.”

Response: Replaced “intermediate host” with “accidental intermediate hosts” as per reviewer’s comments. See line 66 of the revised manuscript.

Line 70: replace “Oxfendazole” with “oxfendazole”

Response: Replaced “Oxfendazole” with “OXF” as per reviewer’s comments. See line 75 of the revised manuscript.

Line 82: replace “ELISA” with “enzyme linked immunosorbent assay (ELISA)”

Response: The "ELISA" has been replaced with "enzyme linked immunosorbent assay (ELISA)" as suggested by the reviewer. See line 87-88 of the revised manuscript.

Line 108: to define "CK-MB"

Response: CK-MB has been defined as creatine kinase -myocardial band (CK-MB). See line 113 of the revised manuscript.

Lines 126-132: An animal ethics protocol (IACUC), not an IRB, is typically required for conducting experiments with animals. If authors have an IACUC protocol, they should include it.

Response: This is not available in Zambia. Therefore, all research be it animal or human are ethically approved by an IRB.

Line 136: replace "Oxfendazole" with "oxfendazole"

Response: replaced "Oxfendazole" with "OXF" as per reviewer's comments. See line number 147 of the revised manuscript.

Lines 156-161: add a reference or references that support the techniques.

Response: References have been added as per reviewer's suggestion to support the techniques. See line 168-173 of the revised manuscript.

Line 181: write *"ad libitum"* in italics

Response: revised as per reviewer's comments to read *"ad libitum"* See line 180 of the revised manuscript.

Line 214: Add oxfendazole information (concentration, brand and country of origin)

Response: The oxfendazole information has been added. See lines 216-217 of the revised manuscript.

Figure 1: replace "T. Solium" with "T. solium"

Figure 1: replace "n= 18" with "n = 18", idem in others numbers

Response: *T. Solium* has been replaced by *T. solium*. See line 155 Fig 1 of the revised manuscript.

REVIEWER #3 (PONE-D-24-05361)

ABSTRACT

- Line 39 – Please show the numbers (mean, sd) of the levels of cTnI in each group

Response: p-values and means of cTnI have been added. See line 39-46 of the revised manuscript.

- Is there any additional information of necropsy? What was the appearance of cyst after treatment? Any inflammatory response in their heart? How did the authors confirm the presence of cysts in the hearts?

Response: Necropsies were conducted at the end of the study. And these have been added in the M&M and Results section. See line 227-234 and 349-367 of the revised manuscript. However, the only limitation was that the level of inflammation was not determined as this required slaughtering of pigs at each sampling point which would not have made any statistical sense in that there were only 9 pigs in each treatment/control group. However, study limitations have been included in the manuscript. See lines 453-457 of the revised manuscript. Moreover, the presence of cysts on tongue examination has been shown by several studies that it is indictive of heavy infection. The heart has been known and proven to be commonly infected in all cysticercosis dissected pigs. This has already been emphasised in the manuscript as it has also been raised by other reviewers**.**

INTRODUCTION

- Line 108 – What does CK-MB stand for?

Response: CK-MB stands for Creatine kinase -myocardial band (CK-MB). See line 113 of the revised manuscript.

M&M

- Line 149 – Could the authors give an estimated prevalence of porcine cysticercosis in the area where pigs came from?

Response: The prevalence of cysticercosis in southern and eastern provinces has been inserted in the text. See line 159-161 of the revised manuscript.

- Line 155 – I am kind of confused with the source of pigs. Authors sought for 18 tongue positive pigs and 18 tongue negative ones from that market; could the authors confirm this? In addition, they said that another group of pigs were purchased from a commercial farm. Please clarify this point.

Response: The tongue positive pigs were purchased from an open market called Chibolya in Lusaka, Zambia and these were 18 in total. Additionally, 18 tongue negative pigs from commercial farms with good health records were included in the study bringing the total number of pigs to 36. However, one pig died in the INT group during the acclimatisation period which reduced the total number to 35.

- Line 166 – what was the purpose of washing and dipping the pigs? If this is not crucial for the development of the trial then it can be removed from the manuscript.

Response: The purpose was to keep the pigs free from dirt as well as ectoparasites such as lice and ticks. But as per reviewer’s comments, this section has been deleted from the manuscript.

- Line 195 – where did the numbers 10 and 20 come from? I would have calculated the sample size using the mean difference of cTnI that was expected in each group.

Response: The numbers 10 and 20 denote the degrees of freedom according to Meads Resource Equation for animal experimental studies, see references (Mead 1988; Festing 2018) in lines 198 and 202 of the revised manuscript.

- What was the mean age of the pigs?

Response: The pigs had an average age of approximately 1 year (12months).

- Line 244 / line 262 – Is there any references for the described technique? I suggest including them as authors might have followed that protocol

Response: The reference for the protocol used has been added. See lines 236 and 238 of the revised manuscript.

- Did the authors perform a necropsy to verify the pig heart infection? It might be possible that the number of cysts in the hearts influence the levels of cTnI.

Response: Necropsies were conducted at the end of the study. And these have been added in the M&M and Results section. See line 227-234 and 349-367 of the revised manuscript. However, the only limitation was that the level of inflammation was not determined as this required slaughtering of pigs at each sampling point which would not have made any statistical sense in that there were only 9 pigs in each treatment/control group. However, study limitations have been included in the manuscript. See lines 453-457 of the revised manuscript.

<u>RESULTS</u>

- Please provide some numbers (means) in this section to be able to visualize those differences

Response: Means and averages have been added in the text as per reviewers' comments. See line 305-318 and 330-336 of the revised manuscript.

- Line 301 – was the "slightly higher" significant? If not, please say it

Response: The slight difference was not significant and this has since been deleted from the text.

- Line 311 – Again, please include some means here to see the differences

Response: Means have been added in the text for visualisation purposes. See lines 330-336 of the revised manuscript.

<u>DISCUSSION</u>

- First paragraph – this is what I would like to see in Results. Instead of SEM add the numerical value

Response: Numerical figures for the SEM have been added in the entire manuscript.

- Line 346 – I think the way it is written is not the appropriate style … "as reported by (17)"

Response: The grammar has been changed to that which is appropriate in the main text.

- Line 359 – was that difference statistically significant compared to non-infected pigs? If not be cautious to rise a conclusion

Response: The difference was not statistically different but may suggest that presence of cysts has an impact on cTnI levels. See lines 400-403 of the revised manuscript.

- Line 338-357 – it is a very long paragraph; it would be better to split into at least two

Response: The referenced paragraph has been divided into two as advised by the reviewer. See line 376-399 of the revised manuscript.

It would be interesting to see what limitations this study presented. For instance, I do not see the necropsy results, number of cysts in the pig hearts, level of inflammation, etc?

Response: Necropsies were conducted at the end of the study. And these have been added in the M&M and Results section. See line 227-234 and 349-367 of the revised manuscript. However, the only limitation was that the level of inflammation was not determined as this required slaughtering of pigs at each sampling point which would not have made any statistical sense in that there were only 9 pigs in each treatment/control group. However, study limitations have been included in the manuscript. See lines 453-457 of the revised manuscript.

REF

- There is a research group which has published several papers on OXF use in pigs, however, I do not see any reference from it. I would suggest to include some of them in the intro or discussion sections.

Response: Two references on oxfendazole use in pigs have been added. See line 134 of the revised manuscript. These have also been added to the reference section.

Funding Statement:

"This study was funded by the German Federal Ministry of Education and Research (BMBF) under CYSTINET-Africa grant number 81203604 (Zambian Chapter) and 01KA1618 (German Chapter). The funder had no role in the design of the study, data collection, analysis and interpretation and in writing the manuscript."

In conclusion, we believe that the revisions we have made have substantially strengthened our manuscript. We are confident that the revised version now meets the high standards of your journal.

Thank you for your time and consideration.

Yours Sincerely

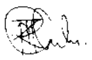

Chibeza Zulu (first/corresponding author)
Email: zuluchibeza1990@yahoo.com
Mobile: +260978348613
