# Supplementary material for: Treatment with oxfendazole increased levels of cardiac troponin I in pigs naturally infected with Taenia solium cysticercosis
Source: PLoS One. 2025 May 8;20(5):e0321735. doi: 10.1371/journal.pone.0321735 (PMC12061117; doi:10.1371/journal.pone.0321735)
Supplement: Attachment — Submitted filename: Response to Reviewers.docx [file pone.0321735.s008.docx]

The University of Zambia	School of Veterinary Medicine	P.O. Box 32379	Lusaka, Zambia

7^th^ February 2025

**The Editor in Chief,**
PLOS One

Dear Sir/Madam,

**REF: RESPONSE TO REVIEWER COMMENTS.**

I am writing to respond to the reviewer's comments on our manuscript, **“Treatment with Oxfendazole increased levels of cardiac troponin I in pigs naturally infected with *Taenia solium* cysticercosis.”** We appreciate the reviewer's thoughtful feedback, which has significantly improved the quality of our work. We have addressed each comment and observation noted by each review as outlined below:

**REVIEWER #4 (PONE-D-24-05361R1)**

**Overall Comments:**

**“It is important for the authors to know and note that cardiac troponin cannot be used as a specific marker for cysticercosis infection in pigs. Cardiac troponin IS a biomarker for any form of cardiac injury and non-specific for injury related to cysticercosis. Therefore, any stress related cardiac injury, or other underlying diseases that cause myocardial injury in pigs will cause the release of cardia troponin. Given this, the fundamental premise of the study is flawed.”**

**“Evaluation of cardiac troponin is not specific, as other cardiac diseases or infection that targets cardiac muscles will induce myocardial cell damage that will release cardiac troponin, so elevation of cardiac troponin level doesn’t necessarily translate to cardiac cysticercosis.”**

**Response:** The basis of this study was on the understanding that animals such as pigs, mice, and guinea pigs for example, have long been used to study various diseases before such studies are done in humans e.g., clinical trials, basic experiments. In this particular case, cardiac troponin I (cTnI) is a biomarker indicative of cardiac damage in humans and animals in various diseases/conditions. Therefore, the study aimed at finding out if cardiac troponin I (cTnI) can also be used to indicate damage to the cardiac muscle in pigs with cysticercosis after treatment. See lines 102 to 110 of the revised manuscript. The basis of the study was also emphasised in the abstract section. See lines 22-27 of the revised manuscript.

Furthermore, pigs positive on tongue examination are heavily infested with cysts in the heart (cardiac muscle), among other organs as shown by several studies and this study as well from the dissection results of the *Taenia solium (T. solium)* positive pigs. Additionally, treatment with an anthelminthic such as oxfendazole (OXF) results in an inflammatory response that causes damage to the surrounding tissue and muscles. This in turn results in an increase in cTnI levels as evidenced from the results in this study. See lines 72-75 of the revised manuscript. References regarding studies that have been conducted in humans and animals on cTnI can be found in the introduction of the manuscript. See lines 111-120 of the revised manuscript.

**“So, there are no gross pictures of cyst-infested tissues from the necropsied animals, even on the last of experiment? Not even the heart that is the basis of this study? No histopathologic evaluation of tissues to support their findings of presence of cysts, inflammation, and mineralization? No evaluation of cytokine levels? No immunohistochemistry?”**

**Response:** Gross pictures from pig dissections of the heart in the IT and INT groups have been inserted. See Fig 7 and Fig 9 of the revised manuscript. Histopathology, estimation of cytokine levels or immunohistochemistry were not done. However, a correlation analysis between cTnI and number of cysts was performed.

**“Does the number of cysts correlate with an increase in cardiac troponin?”**

**Response:** Yes, the increase in cardiac troponin corresponds to the number viable/active cysts. However, this correlation was only significant in the INT group as can be seen from the Pearson correlation analysis results that revealed a strong positive correlation between number of active/viable cysts and concentration of cTnI. See Fig 8 and Fig 10 of the revised manuscript.

**“Carcass dissections at the end of the study also confirmed presence of cysts in the heart.”
No pictures to document this? Do the number of cysts in tissues increase overtime?”**

**Response:** Carcass dissections confirmed the presence of viable/active, degenerated and calcified cysts in both treated and untreated pigs *T. solium* positive pigs i.e., IT and INT. Gross pictures of the carcass dissections have also been included in the manuscript. See Fig 7 and Fig 9 of the revised manuscript. Furthermore, the number of cysts do not increase overtime because these pigs were confined and hence did not pick up any other infection from the environment during the course of the study.

In conclusion, we believe that the revisions we have made have substantially strengthened our manuscript. We are confident that the revised version now meets the high standards of your journal.

Thank you for your time and consideration.

Yours Sincerely

Chibeza Zulu (first/corresponding author)
Email: zuluchibeza1990@yahoo.com
Mobile: +260978348613
